# Antiseizure medication in early nervous system development. Ion channels and synaptic proteins as principal targets

**DOI:** 10.3389/fphar.2022.948412

**Published:** 2022-10-14

**Authors:** Patricio A. Castro, Ingrid Pinto-Borguero, Gonzalo E. Yévenes, Gustavo Moraga-Cid, Jorge Fuentealba

**Affiliations:** ^1^ Laboratory of Physiology and Pharmacology for Neural Development, LAND, Departamento de Fisiología, Facultad de Ciencias Biológicas, Universidad de Concepción, Concepción, Chile; ^2^ Departamento de Fisiología, Facultad de Ciencias Biológicas, Universidad de Concepción, Concepción, Chile

**Keywords:** pregnancy, teratogenicity, neural development, epilepsy, antiseizure medication (ASM)

## Abstract

The main strategy for the treatment of epilepsy is the use of pharmacological agents known as antiseizure medication (ASM). These drugs control the seizure onset and improves the life expectancy and quality of life of patients. Several ASMs are contraindicated during pregnancy, due to a potential teratogen risk. For this reason, the pharmacological treatments of the pregnant Women with Epilepsy (WWE) need comprehensive analyses to reduce fetal risk during the first trimester of pregnancy. The mechanisms by which ASM are teratogens are still under study and scientists in the field, propose different hypotheses. One of them, which will be addressed in this review, corresponds to the potential alteration of ASM on ion channels and proteins involved in relevant signaling and cellular responses (i.e., migration, differentiation) during embryonic development. The actual information related to the action of ASM and its possible targets it is poorly understood. In this review, we will focus on describing the eventual presence of some ion channels and synaptic proteins of the neurotransmitter signaling pathways present during early neural development, which could potentially interacting as targets of ASM. This information leads to elucidate whether these drugs would have the ability to affect critical signaling during periods of neural development that in turn could explain the fetal malformations observed by the use of ASM during pregnancy.

## 1 Introduction

Epilepsy is a chronic pathology that affects near 50 million people globally. Its causes include genetic, structural and metabolic aspects, while in a half of reported cases have an undetermined etiology (Pan American Health Organization, 2018). According to the International League Against Epilepsy (ILAE), this disease is defined as a brain disorder characterized by at least one of three conditions. 1) epileptic syndrome diagnostic, 2) exhibit at least two non-induced seizures in a 24-h range, and 3) present at least a 60% probability of generating a new non-induced seizures during the 10 years after the first two seizures (Fisher et al., 2014).

Treatment for epilepsy tries to contain seizures through pharmacologic management, using a set of drugs called antiseizure medication (ASM). Only 70% of affected people respond effectively to ASM, mostly using monotherapy, but it has been documented that between 20%–30% of all those patients do not respond to pharmacological treatments (Pan American Health Organization, 2018; Fattorusso et al., 2021).

Food and Drug Administration (FDA) and European Medicines Agency (EMA) provide a list containing the drugs approved for use, while the choice and concentration of these ASMs vary for each patient based on factors such as type of epilepsy (syndrome), lifestyle, age, seizure frequency and others. Some of the most frequently ASM used are Phenobarbital (PB), Phenytoin (PHT), Carbamazepine (CBZ) and Valproic acid (VPA), from the first generation drugs. Lamotrigine (LTG), Topiramate (TPM), Levetiracetam (LEV) and Gabapentin (GBP) corresponding to second generation and Lacosamide (LCM), Rufinamide (RUF), Cannabidiol (CBD) between others from third generation (Hakami, 2021).

The malformations rates decreases with the use of third generation ASM associating a more safe profile to the newer drugs (Tomson et al., 2019). Regarding MCM rate, ASM can be classified as low: ≤3% (OXC, GBP, LTG, LEV); intermediate: 3.1%–6% (TPM, CBZ, PHT); high 6.1%–9% (PB); very high > 9% (VPA) (Abou-Khalil, 2022). Despite this, the use of first generation ASM is still broadly use, not only against epilepsy, but is also use for migraine, mood stabilizer and even pain. In addition, the use of ASM can lead to psycho-behavioral side effects and physical dysfunction, such as irritability, sedation, nausea and others (Johnson, 2019).

Fetal malformations include heart defects, cleft palate and failures related to development of the nervous system, such as neural tube defects (NTDs), all of them classified as major congenital Malformations (MCMs) (Källén et al., 1989; Werler et al., 2011; Wallingford et al., 2013). The relationship between MCM and the use of ASM comes mainly from the three registries: NAAPR, UK and Ireland and EURAP. Since these antecedents, the teratogenicity in children of pregnant WWE has been associated especially at the use of ASM in high doses (Pennell, 2016). VPA, have cut-offs for higher risks ranging from 500–650 mg/day. A dose-dependent effect was also identified for LTG, CBZ and PB, while the lowest risk was associated with LTG at ≤325 mg/day (Tomson et al., 2019b). In general, the data shows that elevated MCM rates are associated with the use of high concentrations of VPA and CBZ in comparison with other ASM like LEV (Tomson et al., 2019a). For more detailed information associated with dosage, change in serum levels and bioavailability during pregnancy related with MCM refer to Hakami (2021) and Nucera et al. (2022). In relation with polytherapy, it has been usually considered that multidrug treatments correlate with greater MCMs (Veroniki et al., 2017), nevertheless, more recent studies identify that the specific ASM used is more significant than the number. Once again, the inclusion of VPA was associated with higher prevalence of MCMs (Holmes et al., 2011).

Analyses of teratogenicity in the Central Nervous System (CNS) has been evaluated using frog embryos (*Xenopus laevis*), showing that exposure in early stages of development, such as neurulation, interferes processes related to cell migration and proliferation generating alterations in glutamate signaling (Sequerra et al., 2018). In addition, autism spectrum disorders (ASD) and intellectual disabilities has been associated with the prenatal exposure of ASM (Bjørk et al., 2022).

Although the mechanisms of ASM to control epilepsy, through ion channels or receptors have been extensively studied, the pathways underlying the teratogenicity during intrauterine development are far for complete. Therefore, it is necessary to investigate the possible association between ASM, ion channels, synaptic proteins and teratogenicity during embryonic development.

## 2 Main focus

There is large evidence describing teratogenicity in pregnant WWE with ASM treatment during her first trimester of pregnancy (Kilic et al., 2014; Vossler, 2019). Focused on nervous system development, normally its begins with neurulation process in the first month of pregnancy, followed with a series of complex cellular and tissue modifications such as segmentation, migration, differentiation, axonal guidance, synaptogenesis, among others (Knuesel et al., 2014). Based on this information, we could hypothesize that the generation of some neural teratogen alterations would occur due to the disturbance of the ASM with active signaling pathways required for aforementioned biological processes.

## 3 Early nervous system development

Neurulation is one of the first step for the development of the nervous system in chordates (Colas & Schoenwolf, 2001; Knuesel et al., 2014). This event is preceded by neural induction (described by Spemann & Mangold on 1924), where a layer of ectodermal cells differentiates and forms the neural plate. This flat layer stretches cephalo-caudally and divides symmetrically while the lateral edges elevate to converge medially and merging to create an internal cavity known as the neural tube. Neurulation takes place in humans during the third week of gestation but its temporary window is specific for each chordates. It is a previous event to synaptogenesis, which occurs around the 20th week of gestation in humans (Knuesel et al., 2014). There is evidence that electrical activity and neurotransmitter signaling is present during neurulation (Root et al., 2008), participating in the regulation of neural plate cell proliferation and migration necessary for the formation of the neural tube (Sequerra et al., 2018; Benavides-Rivas et al., 2020).

Failures in neurulation process leads to NTDs, being anencephaly (erroneous closure of the cranial region) and spina bifida (failure of closure in the caudal zone) (Hughes et al., 2018) the most common malformations. The etiology of NTDs is diverse, involving genetic and environmental factors (Padmanabhan, 2006). Associate to genetic causes, folate deficiency deregulates critical cell remodeling, necessary for this period, like apical constriction (Balashova et al., 2017), while and important environmental factor is the use of ASM during pregnancy (Pippenger, 2003).

Some hypotheses suggests that there is an increase in apoptosis of neural cells results from the interaction of ASM with neurotrophins, NGF and BDNF, interfering with their neuroprotective action (Huang & Reichardt, 2001; Roullet et al., 2010). It is also postulated that the deleterious action of excessive free radicals present in ASM-treated women during pregnancy could be the cause for birth defects (Pippenger, 2003). Other studies argue that the teratogenicity of ASM like valproic acid is related to its known inhibitory action on histone-deacetylase (HDAC), which leads to indirect changes in DNA methylation and gene expression (Eyal et al., 2004; Smith et al., 2012). Here, we will discus the possible teratogen mechanism of ASM through their principal targets.

## 4 General characteristics of ASM

**FIGURE 1:**
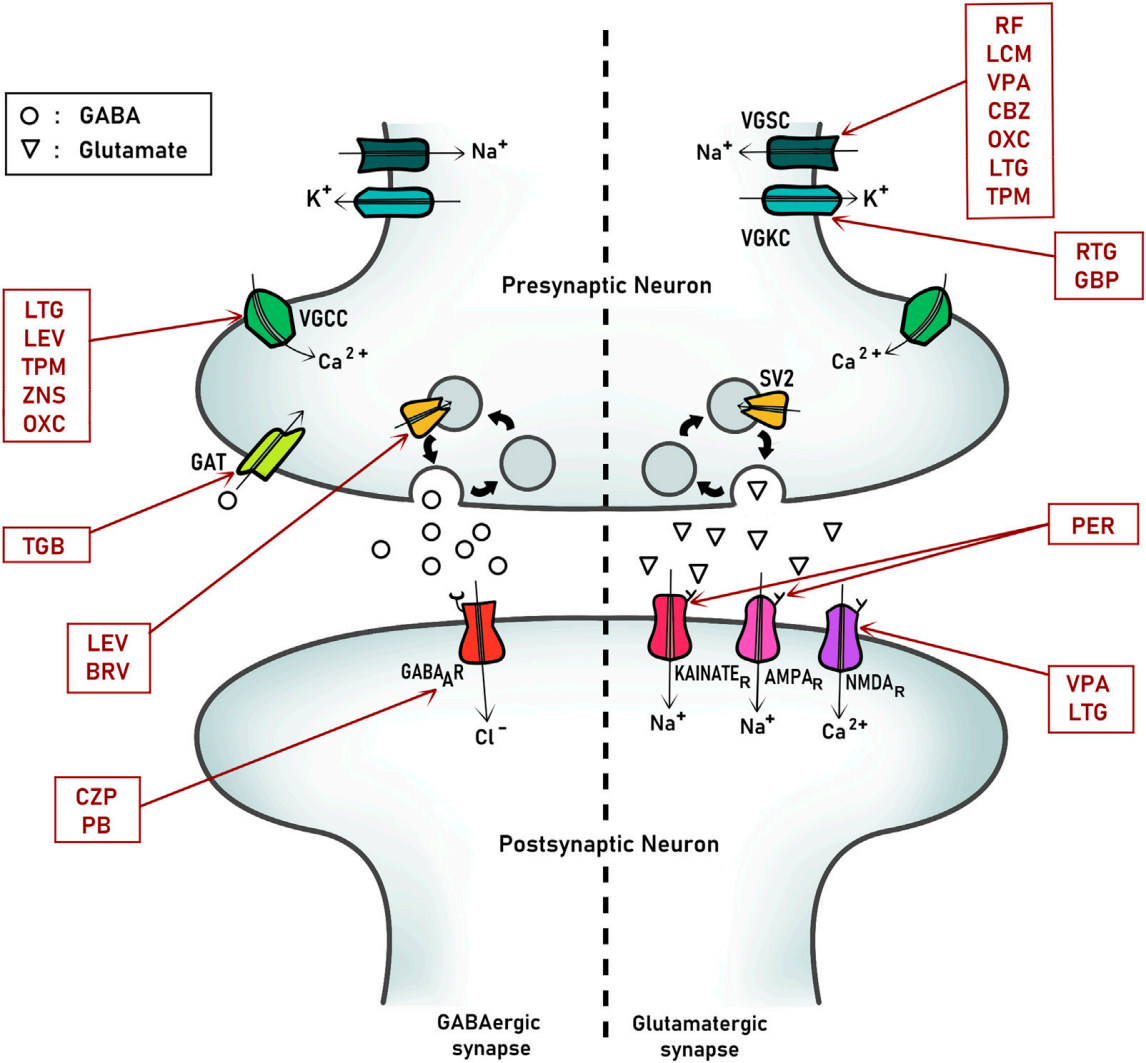
Principal pharmacological targets of antiseizure medication (ASMs) at synapses. A representative scheme of an inhibitory (GABAergic, left) and excitatory (Glutamatergic, right) synapse from a mature nervous system. The main proteins and ion channels targets of relevant antiseizure medications are indicated. VCCC, Voltage-gated calcium channel; VGSC, Voltage-gated sodium channel; VGKC, Voltage-gated potassium channel; SV2, Synaptic-vesicle protein 2. LEV, Levetiracetam; TPM, Topiramate; TGB, Tiagabine; BRV, Brivaracetam; CZP, Clonazepam; PB, Phenobarbital; RF, Rufinamide; LCM, Lacosamide; RTG, Retigabine; GBP, Gabapentin; PER, Perampanel; VPA, Valproate; CBZ, carbazepine; LTG, Lamotrigine; OXC, oxcarbazepine.

ASMs are drugs used to control epilepsy by reducing the frequency or intensity of seizures. It should be noted that these drugs do not modify disease properties, and instead they intended to stabilize its manifestations controlling epileptic seizures. In fact, currently the term use is ASM in replacement of antiepileptic like before. The principal mechanism of action of ASMs is based on controlling the over-excitability of nervous system by modulating ion channels associated with this function, like voltage-gate channels, selectively permeable to Na^+^, K^+^, and Ca ^2+^ and excitatory (glutamate) and inhibitory (GABA) receptors and signaling (Figure 1). In this review, we will focus on describing those proteins that are the principal targets of ASMs to suggest possible interactions between these drugs and embryonic proteins and signaling.

## 5 Channels in early nervous system development

Ion channels allow the passage of Na+, K+, Ca 2 + or Cl-ions, modulating the action potential. These channels can be classified into three broad types depending on the stimulus they need to open or close: 1) mechanosensitive channels, 2) ligand-activated channels, and 3) voltage-dependent channels. The voltage-dependent channels are the target of several ASMs listed below.

### 5.1 Sodium channels

Voltage-gated sodium channels (VGSCs) are composed by one *α* subunit, with genes encoding the proteins Nav1.1 through Nav1.9. They may also have one or two *β* subunits encoding the Navβ1 to Navβ4 proteins. In the adult mammalian central nervous system, four of these α subunits are present: Nav1.1, Nav1.2, Nav1.3, and Nav1.6 (Goldin, 2001; Whitaker et al., 2001).

During rat development, Nav1.1 transcripts are first detected before birth on embryonic day 18 (E18) and their levels increase towards adulthood. Nav1.2 begins to be expressed a little earlier at stage E15 with greater levels detected in the spinal cord peaking at postnatal day 7 (P7) and increasing further in other regions. Nav1.3 is robustly expressed at E12 and decreases thereafter reaching a plateau during P7-P15 (Beckh et al., 1989). Relative expression of Nav1.6 transcripts is quite low in embryonic periods in rats, but it increases early after birth (P1) with development (Schaller & Caldwell, 2000).

Studies show that mutations in the Nav1.1, Nav1.2, Nav1.3, Nav 1.6, and Navβ1 subunits correlate with epilepsy (Guo et al., 2008; Larsen et al., 2015; Wolff et al., 2017) and ASMs are aimed to restore normal ion channel activity altered by these mutant subunits.

A depolarization of plasma membrane generates an Action Potential that in turn is transmitted by axonal VGSCs to further spread the seizure activities. Because of this VGSC are the main targets of several ASM like PHT, CBZ, VPA, LMT, OXC, TPM, ESL, RUF, and LCM. Rufinamide (RF) for example, has reported to have a higher affinity for the Nav1.1 and Nav1.6 subunit proteins (Gilchrist et al., 2014), and Lacosamide (LCM) exerts inhibitory effects on Nav1.3 and Nav1.7 (Sheets et al., 2008). Here, a valid question is if the VGSC signaling is active and participate in early development. Preliminary, the expression levels of VGSC in early stages of neural development (neurulation) would be weak, then, why some ASM with a sodium channel blocker (SCB) action mechanism displays a teratogen risk. Analyses shows that one possible explanation could be its unspecific action. VPA for example, induce ROS formation and apoptosis and inhibit histone deacetylase (HDAC) (Tomson et al., 2016). CBZ, is a potent enzyme inducer acting through 1A2, 2B6, 2C9, 2C19, and 3A4/5 CYP targets acting directly on endogenous metabolic pathways and also has been documented that enhances adipogenesis inhibiting Wnt/β-Catenin expression (Brodie et al., 2013; Lawthom, 2020). PHT, inhibit non-NMDA glutamate receptors with greater affinity to the Ca^2+^-impermeable AMPA receptors (Dron et al., 2021) and additionally inhibit the cardiac calcium release channels ryanodine receptor 2 (RyR2; Ashna et al., 2020). TPM is an antagonist of AMPA and Kainate receptors, increases GABA(R) responses, inhibits carbonic anhydrase isoenzymes, affects voltage-activated Ca^2+^ channels and interact with protein kinase phosphorylation sites (Bai et al., 2022). LMT inhibits postsynaptic AMPA receptors, N- and P/Q-type calcium channels on presynaptic nerve terminals and glutamate release (Dron et al., 2021). Altogether, shows that probably secondary activities of the SCB could contribute significantly to the teratogen risk.

### 5.2 Calcium channels

Voltage-gated calcium channels (VGCCs) are composed of an α1 subunit that detects the potential change, forms the pore, and other auxiliary subunits such as α2δ (encoded by four genes: CACNA2D1-4), β (encoded by four genes: CACNB1-4), and *γ* (encoded by eight known genes CACNGG1-8) (Catterall, 2000). VGCCs are classified according to the activation of its α1 subunit, channels of high conductance (type L, P/Q, N and R) and low conductance (type T). The L-type include the Cav1.1 to Cav1.4 proteins, the P/Q, N, and R types have only one member each, Cav2.1, Cav2.2, and Cav2.3, respectively, while the T-Type contains the Cav3.1 to Cav3.3 subunits.

Associated to nervous system development, Cav2.1 and Cav2.2 are already functional in St.5-6 In *Xenopus laevis* embryos (Motin et al., 2007; Cohen-Kutner et al., 2010), and Cav1.2, Cav2.1, Cav2.2 and Cav3.2 channels are present at St.14 in neural cell cultures. It is important to mention that expression of Cav1.2 disappear at St.18 while Cav1.3 show up only from St.22 (Lewis et al., 2014).

In patients with epilepsy, have been detected alterations in several genes encoded by Cav2.1 (Chioza et al., 2001; Bomben et al., 2016), Cav2.3 (Weiergraber et al., 2006), Cav3.1 (B. Singh et al., 2007), Cav3.2 (Chen et al., 2003; Eckle et al., 2014), and α2δ subunits encoded by the CACNA2D1 and CACNA2D2 genes (Edvardson et al., 2013; Hino-Fukuyo et al., 2015; Vergult et al., 2015).

Levetiracetam (LEV) and Lamotrigine (LTG) have a higher affinity for Cav2.2 (N-) channels (Wang et al., 1996; Lukyanetz et al., 2002). Topiramate (TPM) exerts part of its function on Cav2.2, Cav2.3 channels and L-type channels (Zhang et al., 2000; Kuzmiski et al., 2005), while Zonisamide (ZNS) inhibits T-type channels (Suzuki et al., 1992). ASM have also been reported as therapeutic targets of VGCC complementary subunits, for example, Pregabalin and Gabapentin bind to α2δ helper subunits encoded by the CACNA2D1 and CACNA2D2 genes (Gee et al., 1996; Hendrich et al., 2008).

One study show that use of 200 μM nifedipine a broadly VGCC blocker generates NTDs inhibiting apical constriction of neural plate cells (Suzuki et al., 2017). Other investigation report that neural tube closure signaling pathway require T-type calcium channels (TTCCs) that controls EphrinA expression and loss of TTCCs produces a failure to seal the anterior neural folds, generating NTDs (Abdul-Wajid et al., 2015). These investigations shows that Ca^2+^i is active and relevant during neurulation through VGCC and that alterations on this signaling lead NTDs, like spine bifida.

## 6 Regulation by neurotransmitters in the early development of the nervous system

### 6.1 Excitatory glutamatergic transmission

Glutamate is the main excitatory neurotransmitter of the central nervous system. An aberrant enhancement of glutamatergic neurotransmission can result in epileptic activity. In the nervous system, glutamate receptors are divided into metabotropic (mGluR) whit eight receptors (mGluR1-R8), and ionotropic (iGluR), which are subdivided into three groups: NMDA (containing the GluN1, GluN2A-2D and GluN3A-3B subunits), AMPA (GluA1-GluA4 subunits), and KAINATE (GluK1 to GluK5 subunits). Several receptors have been associated with epilepsy such us: GluA1, GluA2, GluN1, GluN2A, GluN2B, GluK2 and GluK5 (Smolders et al., 2002; Li et al., 2010; Peret et al., 2014; Egbenya et al., 2018; Zubareva et al., 2018).

Several ASMs target glutamatergic-signaling components. Perampanel (PER) is an AMPA receptor antagonist that decreases the affinity of GluA1/2 and GluA2/3 subunit combinations for glutamate (Augustin et al., 2018; Lange et al., 2019). Lamotrigine also inhibits AMPA channels in a dose-dependent manner (Lee et al., 2008) and topiramate inhibits AMPA and KAINATE receptors (Ängehagen et al., 2004).

A study showed that in the neural plate stage of *Xenopus laevis* (St.13) there is glutamate signaling that regulates Ca^2+^ transients through the GluN1 subunit of NMDARs, which it will be a target of the VPA (Sequerra et al., 2018). In addition, the presence of GluA1 receptor transcripts was described in the same development stages, as GluA2 transcripts begin to be expressed in rats at E18 (Qiu et al., 2012) and GluK1 and GluK2 transcripts are present in E17 rats (Joseph et al., 2011). As mentioned early, Ca^2+^ signaling is relevant during neurilation even before and several glutamate-mediated Ca^2+^ receptors like NMDAR and AMPAR will be active at these stages of nervous system development.

### 6.2 Inhibitory GABAergic regulation

The main inhibitory neurotransmitter in the brain is γ-aminobutyric acid (GABA), synthesized from glutamate by the enzymes GAD65/67. GABA receptors can be divided into metabotropic [GABA(B)R] coupled to Gαi protein, which are composed of the B1 and B2 subunits; and ionotropic [GABA(A)R], which allow the selective passage of Cl^−^, composed by varied heteropentameric subunits (α1-6, β1-4, γ1-3, δ, and ρ) (Bettler & Fakler, 2017). A third type of GABA receptor called GABA(A)ρ [also known as GABA(C)R], is a sub-class of the ionotropic GABA(A)R receptor that presents the ρ subunit, and is expressed principally in the retina (Polenzani et al., 1991). In epilepsy, animal models suggest that alterations in GABA(A)R which contain α1, α5 (Friedman et al., 1994; Hernandez et al., 2019), δ (Dibbens et al., 2004; H.-J. Feng et al., 2006), γ2 (Baulac et al., 2001; Eugène et al., 2007), β1 and β3 (Homanics et al., 1997; Brooks-Kayal et al., 1998; Janve et al., 2016) subunits correlate with seizure states.

Before year 2000, studies showed that the α4, β1, γ1 subunits and the GAD65 and GAD67 enzymes were already present in mice at embryonic E17 (Ma & Barker, 1998). Kaeser and colleagues (Kaeser et al., 2011) identified, in stage 8 *Xenopus laevis*, transcripts of the α2 and ρ2 subunits, and transcripts of the ρ1 subunit that decrease by St.16. Levels of transcripts for α1, α3 and β1 increase from St.12-16 and the β2 subunit is detected later at around St.28. Another study in *Lhx6*-eGFP transgenic mice described the presence of transcripts for GABA(A) subunits α1-5, β1-3, γ1-3 at E14.5, (Cuzon Carlson and Yeh, 2011).

Benzodiazepine and barbiturates also has been use as ASM. Clonazepam (CZP) is a positive allosteric modulators to the α-γ2 site of the GABA(A) channel, increasing its opening, (Löscher & Rogawski, 2012; Kubová et al., 2020). Similarly, pentobarbital (PB) are also a positive allosteric modulators of GABA(A)R through their binding to the β3 subunit, binding site different from that of benzodiazepines (Serafini et al., 2000; Zeller et al., 2007; Löscher & Rogawski, 2012). Additionally, PB is considered a high inductor of MCM and has been shows that additional mechanism of action is related to inhibit more selectively the Ca^2+^-impermeable AMPA receptors and N- and L-voltage-activated Ca^2+^ currents (Sills and Rogawski, 2020). A study in pregnant rats where GABA agonists and antagonists were administered, showed that GABA(A)R or GABA(B)R agonists or GABA(B)R antagonist lead to NTDs suggesting a role for GABAergic signaling during neural tube formation (Briner, 2001).

## 7 Other mechanisms of regulation of ASMs: Synaptic vesicles

Synaptic Vesicle Protein 2 (SV2) family are proteins with vesicular localization that participate in neurotransmitter release. In vertebrates, there are three isoforms (SV2A, SV2B, and SV2C) (Bajjalieh et al., 1994; Abdellah et al., 2004; Gregory et al., 2006; Zody et al., 2006). SV2A is the most ubiquitously expressed in the brain, while SV2B has a more restricted expression pattern and SV2C is poorly expressed in the brain, because is highly present in the basal ganglia (Bajjalieh et al., 1994; Janz & Südhof, 1999; Dardou et al., 2011; Crèvecœur et al., 2013; Edvinsson et al., 2015; Steinberg et al., 2016). It has been seen that all these isoforms are closely related to the protein Synaptotagmin, a Ca^2+^ sensor belonging to the SNARE complex, in a binding site inhibited by Ca^2+^, in addition, the SV2A and SV2C isoforms present an additional site of interaction (Schivell et al., 2005).

SV2A knockout mice exhibit a high number of seizures and die by third week of their life, while SV2B knockout animals are viable and do not present severe phenotypic characteristics (Crowder et al., 1999; Janz et al., 1999; Venkatesan et al., 2012). In addition, SV2B levels are decreased in epileptic models and SV2A can be decreased or increased in some epileptic patients (Contreras-García et al., 2018, Contreras-García et al., 2021, Crèvecœur et al., 2014; Feng et al., 2009; Hanaya et al., 2012; Ohno et al., 2009; Shi et al., 2015) which challenges the understanding of the role of SV2 in epilepsy.

The mechanisms by which ASMs might alter the levels or function of these proteins are still under study. The drug Levetiracetam (LEV) exerts its mechanism of action specifically on SV2A proteins (Lynch et al., 2004; Nowack et al., 2011), and a recently developed drug Brivaracetam (BRV), also shows affinity for SV2A, decreasing synaptic frequency and vesicular recycling, presenting a greater affinity for the protein through binding to a distinct site (Yang et al., 2015; Wood & Gillard, 2017).

LEV show a low rate on MCM and the expression of its target proteins during embryogenesis have not been extensively studied, but SV2A is detected as early as in E14 mouse brain (Crèvecœur et al., 2013). Additional mechanism of action of LEV is related with a reduction the amplitude of kainate induced current in cortical neurons (Carunchio et al., 2007) which has not been analyzed during early nervous system development.

## 8 Conclusion

ASMs are the principal treatment for controlling seizures on epileptic patients, but several of these drugs have a secondary effect related with increase the risk of generating MCMs, such as spine bifida (NTDs), which correspond to the first birth defects associate with CNS. ASM target specific channels and receptors related with the control of neuronal excitability (glutamatergic/GABAergic) and then seizure initiation and propagation. Because its association with MCMs, it becomes relevant, describe which of the principal ASM targets proteins are expressed and controls active signaling in early developmental stages of the nervous system. The presence ASM, could interfere with their physiological role and generate birth malformations.

Our analyses identified several investigations, that found the presence of a number of transcripts of voltage-gated channels and receptors in embryonic periods, like Nav1.3 Cav2.1, Cav2.2, GABA(A)α2, and GluA1 which can be target for several ASM like LCM, GBP, RTG, LEV, LTG, TPM, CZP, and PER. One investigation that propose an interaction mechanism between an ASM with an a receptor during neurulation, show that VPA induces NTDs blocking NMDAR and altering excitatory glutamatergic signaling in embryos necessary for generates Ca^2+^i transients and in turn, regulate oriented migration of neural plate cells, fundamental for the normal Neural tube closure (Sequerra et al., 2018). Similarly, the direct blockade of active VGCC decreasing Ca^2+^i signaling with nifedipine (Suzuki et al., 2017) or inhibition of T-Type Ca^2+^ Channel directly generates NTDs, because alter active and necessary signaling for these periods.

The interaction between ASM and its principal target, interfering with an active signaling important for early development, correspond to first possibility of generate malformations of the nervous system. An additional hypothesis is related with the action of ASM and secondary targets. Almost all ASM, interact with additional proteins different to the principal targets (Table 1) and the possibility to interfere with signals different to Na^+^ channels, Ca^2+^ channels, glutamate and GABA receptors and synaptic vesicles proteins (SV2) increases significantly. For example, VPA present at least four targets besides Na^+^ channel, including HDAC, ROS generation, TCA enzymes and GABAergic system. Similarly, TPM affects Na^+^ channels, GABA augmentation, AMPAR and KaiRs. Then, exist a good association between drugs development and safety profile, whose older drugs (first generation) are more unsafe that new (third generation). In correlation, first ASM have more targets possible versus new drugs, restricting the alterations of multiple signaling.

**TABLE 1:** Teratogenic and pharmacological mechanism of action of anti-seizure medication.

Anti-seizure medication, ASM	Major malformation rate^a^	Molecular target^b^	Others molecular targets^c^	Use in WWE^d^
Phenobarbital, PB	High	(+) GABA(A)R	SCB, (-) NMDAR	Avoid
Phenytoin, PHT	Intermediate	SCB	(-) AMPAR, (-) RyR2	Avoid
Valproate, VPA	Very high	SCB	(+) GABA transmission, (-) HDAC, (-) TCA enzymes, (-) NMDAR	Avoid
Carbamazepine, CBZ	Intermediate	SCB	(+) GABA(A)R conductance, (-) Wnt/β-Catenin expression, adipogenesys, modulation purinergic and serotonergic transmission	With caution
Oxcarbazepine, OXC	Low	SCB	(-) Voltage-activated calcium currents	With caution
Lamotrigine, LTG	Low	SCB	(-) N- and P/Q-type Ca^2+^ channels, (-) AMPAR	Recommend
Topiramate, TPM	Intermediate	SCB	(+) GABA(A)Rs, (-) AMPAR/KaiRs, (-) Carbonic Anhydrase, VGCC, PK phosphorylation	With caution
Levetiracetam, LEV	Low	SV2	(-) KaiRs	Recommend
Lacosamide, LCM	Unknown	SCB	Carbonic anhydrase (probably)	Insufficient data

a Data are extracted from North American and European registries, Abou-Khalil BW, 2019.

b Extracted from Nucera et al., 2022.

c Extracted from Sills and Rogawski, 2020; Nucera et al., 2022; Hakami, 2021; Lawthom, 2020; Tomson et al., 2016; Dron et al., 2021 and Stefani et al. (1995).

d Extracted from Nucera et al., 2022.Abbreviations: WWE, women with epilepsy; (+), activator; (−), inhibitor; SCB, sodium channel blocker; NMDAR, N-methyl-D-aspartate receptor; AMPAR, α-amino-3-hydroxy-5-methyl-4-isoxazolepropionic acid receptor; RyR2, ryanodine receptor 2; HDAC, Histone deacetylases; TCA, tricarboxylic acid cycle; GABA(A)R, gamma-aminobutyric acid receptor type A; KaiR, Kainate receptor; VGCC, voltage gated calcium channel; PK, protein kinase.

Despite the development of new ASM, investigations of third generation ASM can generate nervous system malformations *in vitro*. LCM and its metabolites may have teratogenic effects on the developing mice embryos, reflected in embryonic lethality and malformations, as well as behavioral and histological alterations (López-escobar et al., 2020). Then, LCM generates growth retardation and major malformations increased in a dose-dependent manner and observed mostly in the supratherapeutic group (Mete et al., 2016). These preclinical data will need to be corroborated with new investigations and clinical studies, which should confirm the potential risk of using LCM and third generation ASMs.

In summary, the expression of diverse channels and receptors in early stages of development should be associate with a functional role during embryogenesis. The comprehensive knowledge of the function of these components as possible targets of ASMs will help to evaluate possible interactions during intrauterine gestation in pregnant WWE. More studies are needed to determine if these interactions occurs *in vivo*, in order to contribute to the understanding the teratogenic effect of old and new ASM during pregnancy. Finally, in relation with the pathology of epilepsy and seizure onset, it has been shown that the expression of several receptors and ion channels changes with epileptic seizures (Bender et al., 2003) and could be a relevant strategy and target for future analyses of ASM.
